# Age-related Changes in p56^lck^ Protein Levels and Phenotypic Distribution of T Lymphocytes in Young Rats

**DOI:** 10.1080/17402520400017393

**Published:** 2005-03

**Authors:** Heather J. Hosea, Edward S. Rector, Carla G. Taylor

**Affiliations:** 1 Departments of Human Nutritional Sciences University of Manitoba 190 Dysart Road Winnipeg MB R3T 2N2 Canada; 2 Department of Immunology University of Manitoba Winnipeg MB R3E 0W3 Canada

## Abstract

p56^lck^ is involved in the maturation of T-cells from double negative (DN) into double positive (DP) T-cells. The objective of this experiment was to determine changes in the levels of thymic and splenic T-cell p56^lck^ using Western immunoblotting, along with the proportion and number of T-cell subsets in thymus, spleen and blood using flow cytometry in growing Sprague-Dawley rats. Thymic p56^lck^ levels were negatively correlated with age (*r*=-0.42, *p*=0.04) and positively correlated with age in the spleen (*r*=0.50, *p*=0.01). Nine-week-old rats had a higher percentage of thymic DN and CD8 cells with fewer DP cells compared to younger rats. There were minor differences in the proportions of T-cell subsets in the spleen and blood. T-cell numbers remained proportional to body weight in the lymphoid organs; however, the lower absolute number of T-cells in the younger rats might indicate that they are less able to respond to antigens.


*Clinical & Developmental Immunology*, March 2005; 12(1): 75–84

Taylor & Francis

Taylor & Francis Group

### Age-related changes in p56$^{lck}$ protein levels and phenotypic distribution of T lymphocytes in young rats

HEATHER J. HOSEA[1], EDWARD S. RECTOR[2], & CARLA G. TAYLOR[1]

[1]*Departments of Human Nutritional Sciences, University of Manitoba, H507 Duff Roblin Building, 190 Dysart Road, Winnipeg, MB, Canada, R3T 2N2, and* [2]*Department of Immunology, University of Manitoba, Winnipeg, MB, Canada, R3E 0W3*

**Abstract**
p56$^{lck}$ is involved in the maturation of T-cells from double negative (DN) into double positive (DP) T-cells. The objective of this experiment was to determine changes in the levels of thymic and splenic T-cell p56$^{lck}$ using Western immunoblotting, along with the proportion and number of T-cell subsets in thymus, spleen and blood using flow cytometry in growing Sprague-Dawley rats. Thymic p56$^{lck}$ levels were negatively correlated with age ($r = -0.42, p = 0.04$) and positively correlated with age in the spleen ($r = 0.50, p = 0.01$). Nine-week-old rats had a higher percentage of thymic DN and CD8 cells with fewer DP cells compared to younger rats. There were minor differences in the proportions of T-cell subsets in the spleen and blood. T-cell numbers remained proportional to body weight in the lymphoid organs; however, the lower absolute number of T-cells in the younger rats might indicate that they are less able to respond to antigens.

**Keywords:** *Blood T lymphocytes, flow cytometry, p56$^{lck}$, rats, spleen T lymphocytes, thymus*

### Introduction

The immune system is complex and is affected by numerous environmental and genetic factors. Inbred, transgenic and knock-out mouse strains have been invaluable tools for advancing our knowledge of the immune system (Totey and Kukarni 2000, Ravirajan and Isenberg 2002, Wong and Wen 2003). However, the rat is also an important model in experimental biology, and the emerging importance of the immune system in various disciplines and diseases requires a basic understanding of developmental changes in the immune system of the rat. There are greater opportunities to investigate cell signaling and the immune system in rats due in part to the increasing availability of commercial antibodies for rats. The larger size of the rat provides more tissue/cells than the mouse for multiple measurements to explore the relationships between the immune system and physiological/pathological phenomena in the same animal.

The growth stage is a critical point in the development of the immune system and is vulnerable to many environmental and genetic factors. During growth, the bone marrow (stem cell production) and thymus (T cell maturation) are maturing, and they are also responsible for production of the cells involved in the adaptive immune response. T cell function has been shown to be affected by such factors as malnutrition (Woodward 1998), disease (Kannagi et al. 2000, Shou et al. 2001) and drugs (Colombo et al. 1999) and these effects are amplified during growth. As T cells mature in the thymus, their cell surface expression of CD4 and CD8 changes from double negative ($CD4^-CD8^-$) to double positive ($CD4^+CD8^+$) and eventually into single positive T cells ($CD4^+CD8^-$ or $CD4^-CD8^+$) before being released into the periphery (Zamoyska et al. 2003). The signaling protein p56$^{lck}$, which is principally expressed in T lymphocytes, is involved in the maturation of T cells

Correspondence: C. G. Taylor, Department of Human Nutrinational Sciences, H507 Duff Roblin Bldg, 190 Dysart RD, University of Manitoba, Winnipeg, MB, Canada, R3T 2N2. Tel: 1 204 474 8079. Fax: 1 204 474 7593. E-mail: ctaylor@ms.umanitoba.ca

ISSN 1740-2522 print/ISSN 1740-2530 online © 2005 Taylor & Francis Group Ltd.
DOI: 10.1080/17402520400017393

from double negative into double positive T cells (Abraham et al. 1991, Molina et al. 1992, Levin et al. 1993, Zamoyska et al. 2003). The role of $p56^{lck}$ in thymocyte maturation has been investigated using transgenic animal models and cell culture methods (Glaichenhaus et al. 1991, Sohn et al. 2001). Given the changes in $p56^{lck}$ due to dietary deficiencies (Lepage et al. 1999, Hosea et al. 2003), an environmental factor, it is of interest to determine if any differences exist in the protein levels of $p56^{lck}$ along with the proportion and number of the T cell subsets during growth in rats. Thus, the objective of the present experiment was to determine $p56^{lck}$ protein levels using Western immunoblotting and to characterize the T cell subsets in the thymus, spleen and blood of 3-, 6- and 9-week-old rats using flow-cytometry. Flow-Count™ Fluorospheres were used to determine absolute numbers of the T lymphocyte subsets. Three- to nine-week-old rats represent the weanling phase to sexual maturity, and it takes approximately 3 weeks for a T cell to mature upon entering the thymus (Sharon, 1998).

### Materials and methods

### *Animals*

Three-week-old male Sprague-Dawley rats (Charles River Laboratories, St Constant, PQ) were fed a modified AIN-93G diet (Reeves et al. 1993, Lepage et al. 1999) *ad libitum*. The rats were maintained in an environment of controlled temperature (21–23°C), humidity (55%) and light cycle (14 h light/10 h dark). Animal care was provided in accordance with a protocol approved by the Local Animal Care Committee (University of Manitoba).

### *Tissue collection*

At 3, 6 and 9 weeks of age, rats were killed by $CO_2$ asphyxiation and cervical dislocation. Trunk blood was collected in EDTA vacutainer tubes (Becton Dickinson, Franklin Lakes, NJ). The thymus and spleen were removed and processed immediately.

### *Cell preparation*

Single cell suspensions of spleen and thymus in phosphate buffered saline (PBS)/1% bovine fetal calf serum (Gibco, Grand Island, NY) were prepared using a loose fitting Kontes glass/glass homogenizer (Kontes, Vineland, NJ). Spleen, but not thymus, cell suspensions were gently layered over Lympholyte-Rat (Cedarlane, Hornby, ON) and centrifuged at 1500*g* for 20 min at room temperature to reduce presence of polymorphonuclear cells and erythrocytes. Spleen cells from the interphase layer (primarily mononuclear cells) and thymus cell suspensions were washed twice (400*g* for 10 min) and resuspended at a concentration of $1 \times 10^7$/ml in PBS/5% fetal calf serum.

### *Determination of T Lymphocyte subpopulations*

*Antibodies*. Monoclonal antibodies for TCRαβ (PE label, R73 clone), CD4 (PC5 label, OX-35 clone) and CD8 (FITC label, G28 clone) were obtained from BD Pharmingen (Mississauga, ON). The sample combinations that were used for three-color analysis were: TCRαβ, CD4, CD8 or their respective isotype controls.

*Cell labeling*. Thymus and spleen cells ($1 \times 10^6$) were incubated with the mixture of monoclonal antibodies for 40 min at 4°C. After, addition of 3 ml cold PBS/0.5% bovine serum albumin, cells were centrifuged (300*g* for 6 min), resuspended in a fixation solution of 1% paraformaldehyde in PBS (pH 7.2) and kept on ice. Whole blood (100 μl) was incubated with the appropriate antibodies or isotype controls for 15 min, mixed with 500 μl Optilyse C (Beckman, Mississauga, ON), and subsequently prepared according to manufacturers instructions. FlowCount™ fluorospheres (100 μl, Beckman, Mississauga, ON) were added to tubes for thymus, spleen and blood prior to analysis. Samples were analyzed using a Beckman Coulter EPICS ALTRA (Beckman Coulter Canada, Mississauga, ON) high speed cell sorter with laser excitation tuned to 488 nm (65 mW). Representative flow cytometry plots of cells isolated from the thymus, spleen and blood of a 6-week-old rat are shown in Figures 1–3, respectively. Forward vs. side scatter histograms were used to gate on intact lymphoid cells. Further, gating on cell surface markers is indicated in the tables and figures. The fluorescence signals were separated with the standard dichroic long pass filters provided with the instrument and detected through 525 nm (FITC), 575 nm (PE) and 675 nm (PC5) bandpass filers, respectively. The data were collected in listmode format with the subsequent analyses based on 10,000 cells satisfying the light scatter gate using the EXPO32 MultiCOMP MFA software provided with the instrument. Fluorochrome-istotype matched controls were prepared to assess autofluorescence and nonspecific binding, while single color samples were employed to adjust color compensation. Absolute cell counts were calculated based on the total number of cells counted, the total number of fluorospheres counted and the concentration of the fluorospheres. Cells counts were corrected for the weight of the thymus or spleen used to prepare the cell suspensions.

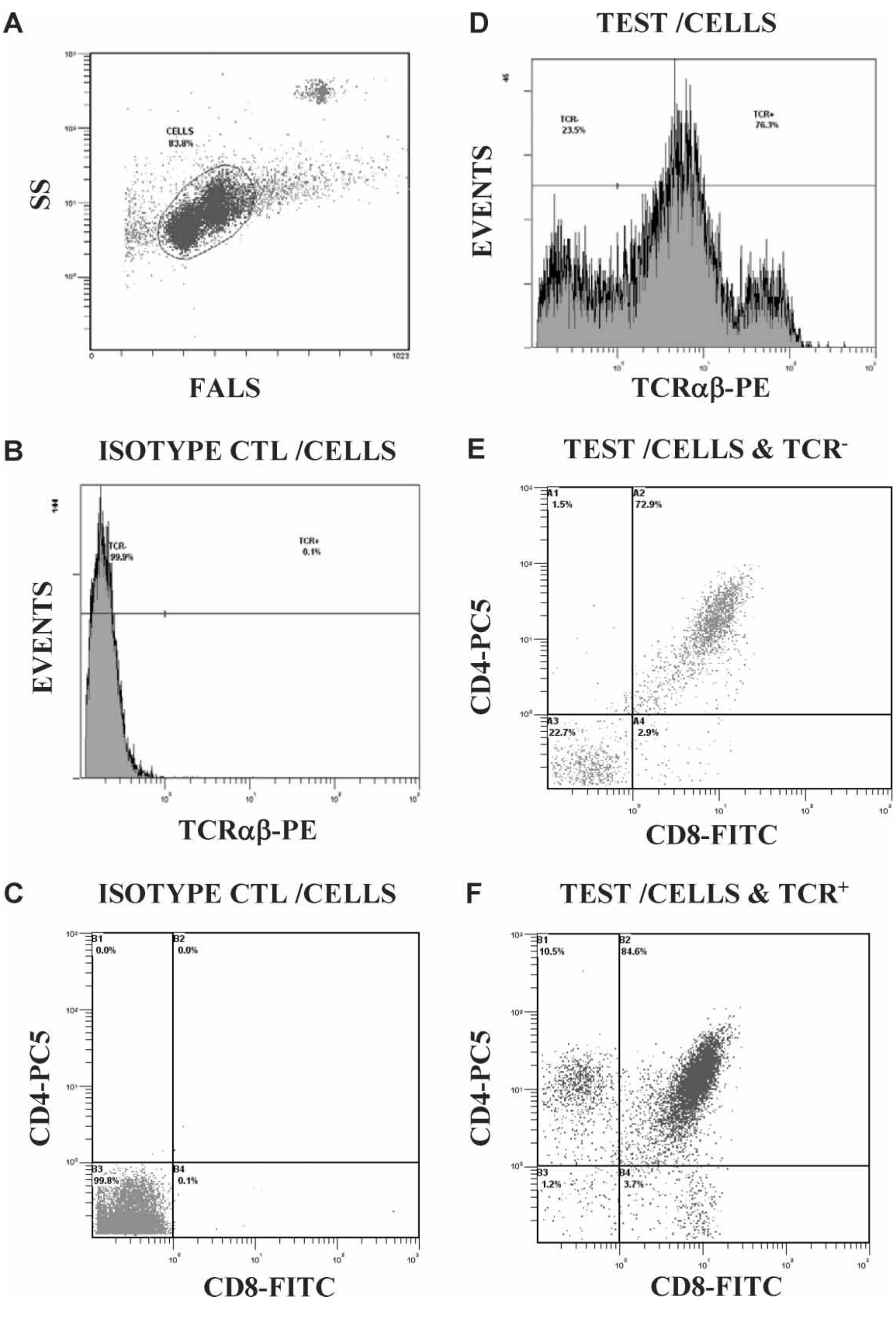

Figure 1. Representative flow cytometry plots of lymphocytes from the thymus of 6-week-old rats. Definition of cell sample (lymphocytes) by light scatter (A); isotype control sample gated on CELLS (B and C); test sample gated on CELLS defining TCRαβ binding (D); test sample gated on CELLS and TCRαβ$^-$ defining CD4$^+$ and CD8$^+$ TCRαβ$^-$ lymphocytes (E); test sample gated on CELLS and TCRαβ$^+$ defining CD4$^+$ and CD8$^+$ TCRαβ$^+$ lymphocytes (F). Abbreviations: SS, side scatter; FALS, forward angle light scatter; CTL, control.

### *Western immunoblotting for p56$^{lck}$*

Cell lysates were prepared by resuspending thymocytes and splenocytes in RIPA buffer containing protease inhibitors as previously described (Lepage et al. 1999). Protein concentration was determined using the BCA Protein Assay (Sigma, St Louis, MO). For Western blotting, cell lysates (20 μg protein per lane), molecular weight standard and positive control (Jurkat Cells Lysate, clone: Human T-cell leukemia, BD Pharmingen, Mississauga, ON) were separated by SDS-PAGE (5% stacking gel and 10% separating gel)

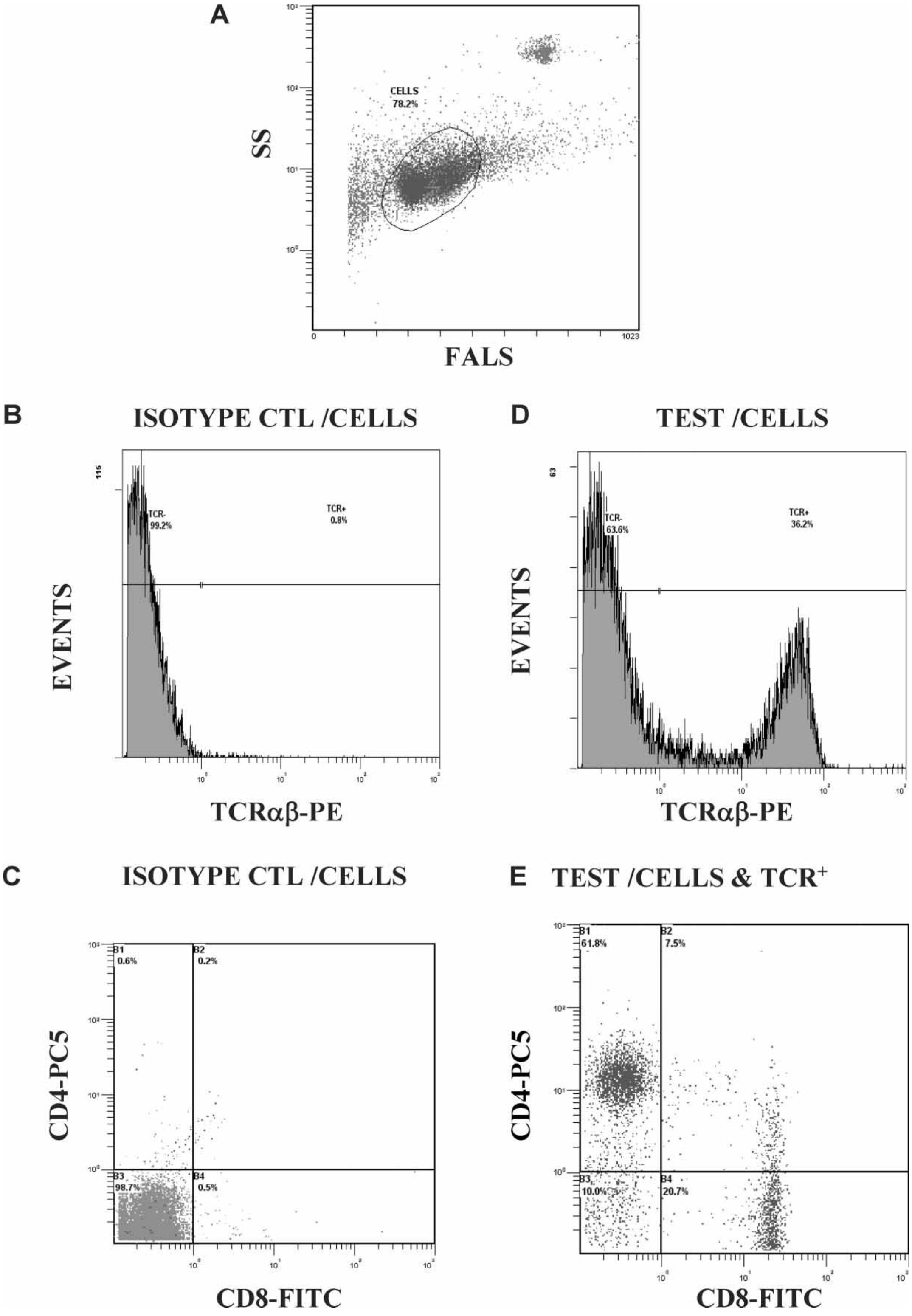

Figure 2. Representative flow cytometry plots of lymphocytes from the spleen of 6-week-old rats. Definition of cell sample (lymphocytes) by light scatter (A); isotype control sample gated on CELLS (B) and CELLS and $TCR\alpha\beta^+$ (C); test sample gated on CELLS defining $TCR\alpha\beta$ binding (D); test sample gated on CELLS and $TCR\alpha\beta^+$ defining $CD4^+$ and $CD8^+$ $TCR\alpha\beta^+$ lymphocytes (E). Abbreviations: SS, side scatter; FALS, forward angle light scatter; CTL, control.

and transferred to nitrocellulose membrane (0.2 um; BioRad, Hercules, CA) using previously published procedures (Lepage et al. 1999). $p56^{lck}$ was detected using mouse anti-human lck (1:5000; clone 28, Transduction Laboratories, Lexington, KY), goat anti-mouse IgG horseradish peroxidase (1:1000) and Chemi Glow (Fisher, Whitby, ON) as the luminescent substrate. Arbitrary

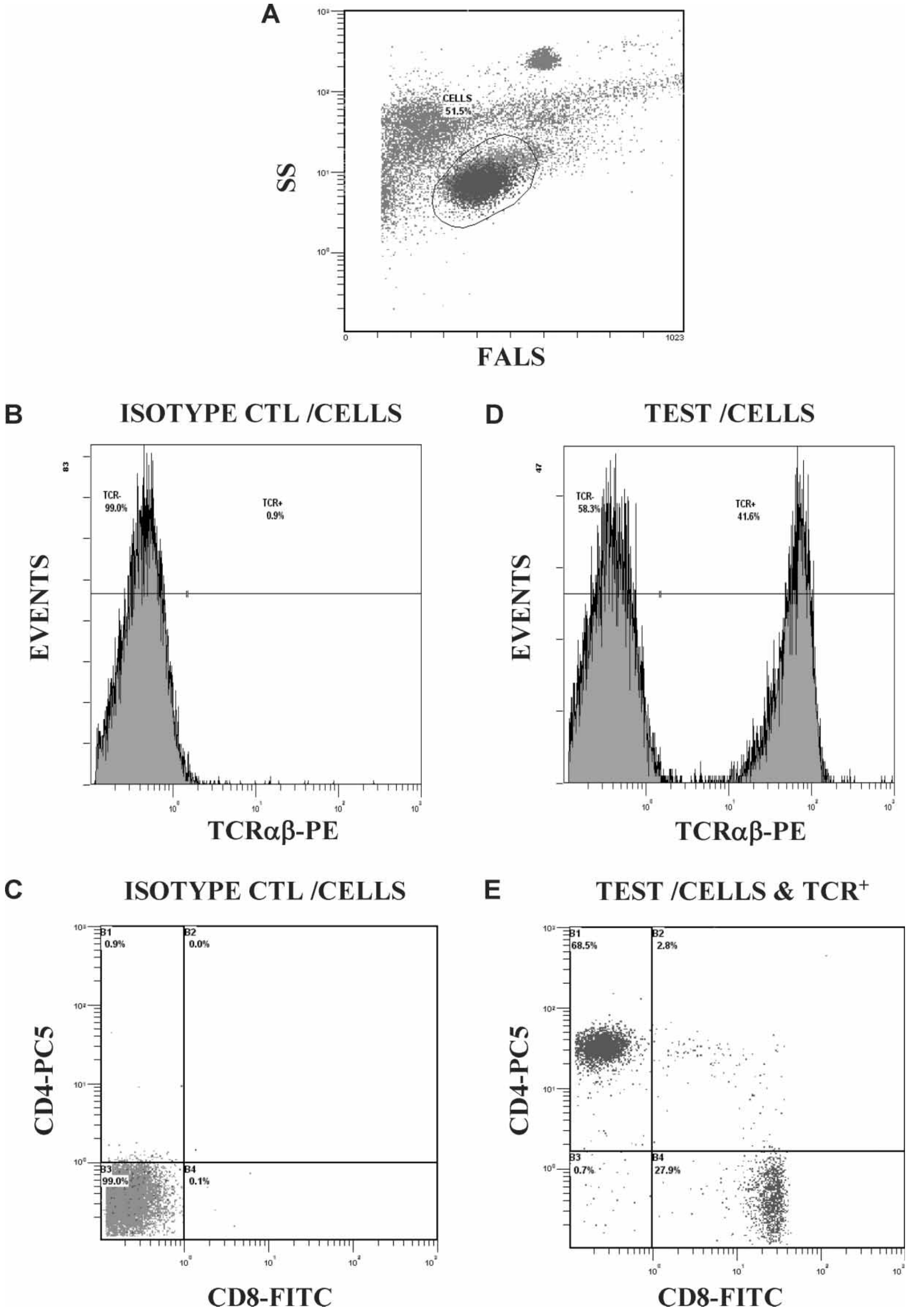

Figure 3. Representative flow cytometry plots of lymphocytes from the blood of 6-week-old rats. Definition of cell sample (lymphocytes) by light scatter (A); isotype control sample gated on CELLS (B and C); test sample gated on CELLS defining TCRαβ binding (D); test sample gated on CELLS and TCR$\alpha\beta^+$ defining $CD4^+$ and $CD8^+$ TCR$\alpha\beta^+$ lymphocytes (E). Abbreviations: SS, side scatter; FALS, forward angle light scatter; CTL, control.

units for bands were determined using the FluorChem digital imaging system (Alpha Innotech Corporation, San Leandro, CA) and FluorChem software (version 2.0).

*Statistical analysis*

Data were analyzed by one-way ANOVA using the general linear models procedure (SAS software release 8.2, SAS Institute, Cary, NC). When necessary, data were normalized by log transformation for statistical analyses, but non-transformed means are reported. Significant differences among means were determined using Duncan's multiple range test. Differences were considered significant at $p < 0.05$. Pearson's correlation coefficient was used to examine correlations between $p56^{lck}$ and age.

### Results

*Body and lymphoid organ weights*

Body weight of 3-week-old rats increased 60% by 6 weeks, and another 30% by 9 weeks of age (Table I). Thymus and spleen weights increased 44% between 3 and 6 weeks, but remained similar between 6 and 9 weeks. Thymus and spleen weights corrected to body weight decreased with age.

*$p56^{lck}$*

$p56^{lck}$ protein levels were negatively correlated with age in the thymus ($r = -0.42, p = 0.04$) and positively correlated with age in the spleen ($r = 0.50$, $p = 0.01$) of 3–9-week-old growing rats (Figure 4).

*$TCR\alpha\beta^+$ lymphocytes*

There were no differences in the proportion of $TCR\alpha\beta^+$ cells (gated on lymphocytes) among age groups in the thymus; however, there was a lower percentage $TCR\alpha\beta^+$ cells in the spleen and blood of 3-week-old rats compared to both 6- and 9-week-old rats (Table II). Three-week-old rats had fewer $TCR\alpha\beta^+$ cells/g of spleen and μl of blood, and per spleen and total blood volume compared to

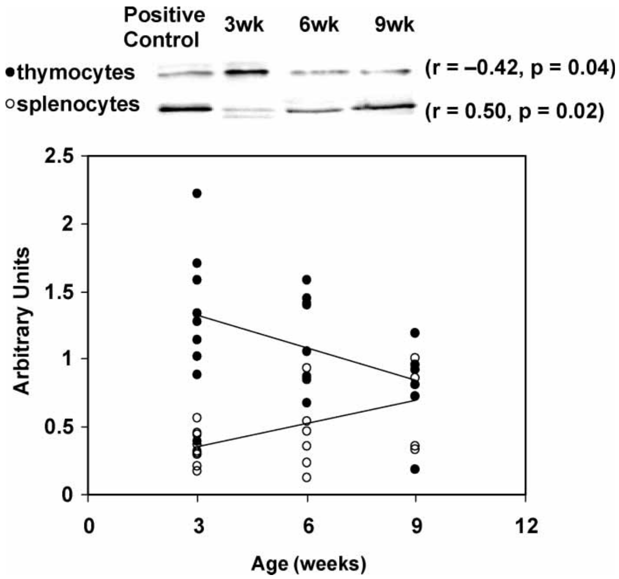

Figure 4. Representative blots and correlations of age with $p56^{lck}$ protein levels in thymocytes and splenocytes of young rats. Data points are values from individual rats ($n = 9, 8, 6$ for 3, 6 and 9weeks, respectively).

both 6- and 9-week-old rats, but no differences were detected among groups in the thymus.

*T Lymphocyte numbers corrected for body weight*

There were no differences among the age groups in the number of thymic or splenic $TCR\alpha\beta^+$ lymphocytes/g of body weight, however, 3-week-old rats had fewer $TCR\alpha\beta^+$ cells/g body weight in their blood compared to 6- and 9-week-old rats (Figure 5).

*Proportions of T lymphocyte subpopulations*

*Thymus*. Figure 6a and b represents the proportion of T lymphocyte subsets in the thymus after gating on $TCR\alpha\beta^-$ and $TCR\alpha\beta^+$ cells, respectively. The proportion of the most immature T lymphocyte phenotype in the thymus ($TCR\alpha\beta^-CD4^-CD8^-$) was lower in 6-week-old rats compared to 9-week-old rats. Both 3- and 6-week-old rats had a lower proportion of $TCR\alpha\beta^-CD4^-CD8^+$ and a higher percentage of

Table I. Body and lymphoid organ weights of 3-, 6- and 9-week-old rats.

| | Age | | |
|---|---|---|---|
| | 3 weeks | 6 weeks | 9 weeks |
| Body weight, g | $116 \pm 2^c$ | $289 \pm 6^b$ | $407 \pm 21^a$ |
| Thymus weight, g | $0.50 \pm 0.03^b$ | $0.90 \pm 0.06^a$ | $0.74 \pm 0.06^a$ |
| Thymus/body weight, % | $0.43 \pm 0.02^a$ | $0.31 \pm 0.02^b$ | $0.19 \pm 0.01^c$ |
| Spleen weight, g | $0.42 \pm 0.02^b$ | $0.74 \pm 0.04^a$ | $0.72 \pm 0.06^a$ |
| Spleen/body weight, % | $0.36 \pm 0.02^a$ | $0.25 \pm 0.01^b$ | $0.18 \pm 0.01^c$ |

Values are mean ± SEM for $n = 9$ (3 weeks), $n = 8$ (6 weeks) and $n = 6$ (9 weeks). Different superscript letters indicate significant differences among means, $p < 0.05$.

Table II. TCRαβ+ expression on lymphocytes from the thymus, spleen and blood of 3-, 6- and 9-week-old rats

| | Age | | |
|---|---|---|---|
| | 3 weeks | 6 weeks | 9 weeks |
| *Thymus* | | | |
| TCRαβ+ | | | |
| % | 71.34 ± 1.51 | 74.38 ± 2.11 | 68.23 ± 2.57 |
| Cells/g ( × $10^7$) | 105.4 ± 45.8 | 100.4 ± 10.2 | 109.9 ± 19.2 |
| cells/thymus ( × $10^7$) | 51.96 ± 25.3 | 90.44 ± 10.8 | 76.86 ± 13.7 |
| *Spleen* | | | |
| TCRαβ+ | | | |
| % | 19.27 ± 2.45[b] | 33.94 ± 1.00[a] | 36.70 ± 2.68[a] |
| cells/g ( × $10^6$) | 18.92 ± 5.08[b] | 46.49 ± 6.17[a] | 47.30 ± 6.94[a] |
| cells/spleen ( × $10^6$) | 8.25 ± 2.44[b] | 35.14 ± 5.69[a] | 32.24 ± 4.27[a] |
| *Blood** | | | |
| TCRαβ+ | | | |
| % | 28.86 ± 1.95[b] | 43.11 ± 2.36[a] | 48.62 ± 2.10[a] |
| cells/μl | 1975 ± 92[b] | 4177 ± 428[a] | 4283 ± 299[a] |
| cells/total blood volume ( × $10^6$) | 12.98 ± 0.62[c] | 69.47 ± 7.32[b] | 97.99 ± 9.58[a] |

Values are mean ± SEM for $n = 9$ (3 weeks), $n = 8$ (6 weeks) and $n = 6$ (9 weeks). Different superscript letters indicate significant differences among means, $p < 0.05$. Absolute cell numbers calculated using FlowCount™ fluorospheres. Percentages and absolute numbers determined after gating on total lymphocytes.
* Total blood volume estimated using 57.5 ml/kg (Olfer *et al.*, 1993).

TCRαβ−CD4+CD8+ cells (the next phenotypes in T lymphocyte maturation) compared to 9-week-old rats. The next step in maturation is TCRαβ+CD4+CD8+ and 3-week-old rats had a higher percentage of TCRαβ+CD4+CD8+ cells, compared to 9-week-old rats. The most mature T lymphocytes found in the thymus are either TCRαβ+CD4+CD8− or TCRαβ+CD4−CD8+. There were no differences among the age groups in the proportion of TCRαβ+CD4+CD8− cells, however, the proportion of TCRαβ+CD4−CD8+ cells was lower in the 3-week-old rats compared to 6- and 9-week-old rats.

*Spleen.* Figure 6c represents the proportion of T lymphocyte subsets in the spleen after gating on TCRαβ+ cells. The percentages of TCRαβ+CD4+CD8+ and TCRαβ+CD4+CD8− cells were lower in the 3-week-old rats compared to the 9-week-old rats, while there were no differences among the age groups in the percentages of TCRαβ+CD4−CD8− or TCRαβ+CD4−CD8+ cells.

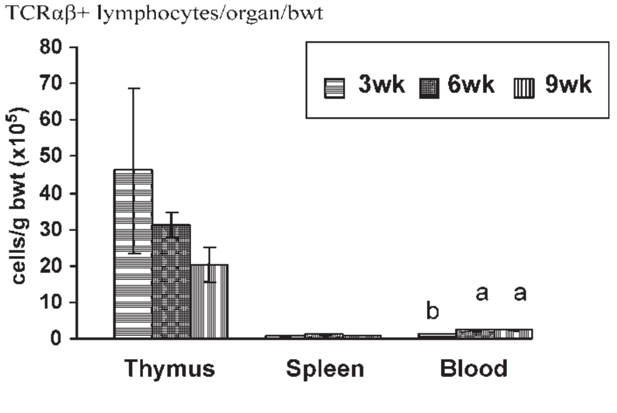

Figure 5. Absolute number of lymphocytes that positively express TCRαβ corrected for body weight. Values are mean ± standarderror for $n = 9$ (3 weeks), $n = 8$ (6 weeks) and $n = 6$ rats (9 weeks). Different superscript letters indicates statistical differences ($p < 0.05$) among age groups.

*Blood.* Figure 6d represents the proportion of T lymphocyte subsets in the blood after gating on TCRαβ+ cells. The percentage of TCRαβ+CD4+CD8− cells was lower in 3- and 6-week-old rats compared to 9-week-old rats, while there were no differences among the age groups in the percentages of TCRαβ+CD4−CD8−, TCRαβ+CD4+CD8+ or TCRαβ+CD4−CD8+ cells.

### *Absolute number of T Lymphocyte subpopulations*

*Thymus.* Figure 7a and b shows the absolute number of T lymphocyte subpopulations in the thymus using FlowCount™ Fluorospheres and gating on TCRαβ− and TCRαβ+ cells, respectively. There were fewer TCRαβ−CD4−CD8− cells in 3-week-old rats compared to 9-week-old rats and fewer TCRαβ+CD4−CD8− cells in 3-week-old rats compared to 9-week-old rats. Three-week-old rats had fewer TCRαβ−CD4−CD8+ and TCRαβ+CD4−CD8+ cells compared to both 6- and 9-week-old rats.

*Spleen.* Figure 7c shows the absolute number of T lymphocyte subpopulations in the spleen

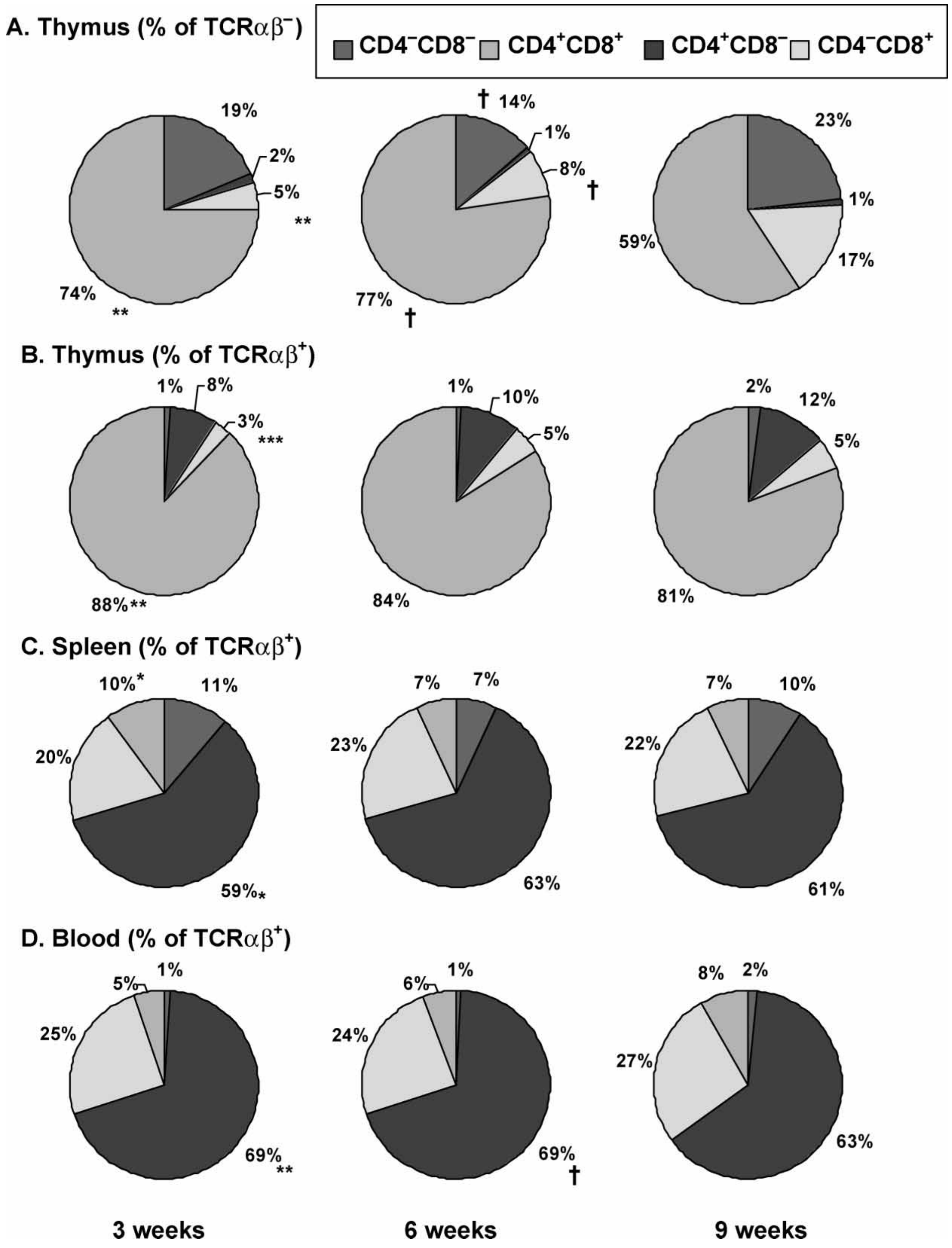

Figure 6. Proportions of T lymphocyte subpopulations in the thymus (a and b), spleen (c) and blood (d) of young rats. Figure 6a was gated on TCRαβ$^-$ cells and figures 6b–d were gated on TCRαβ$^+$ cells. Values are means for $n = 9$ (3 weeks), $n = 8$ (6 weeks) and $n = 6$ rats (9 weeks). Significant differences ($p < 0.05$) among means are indicated by symbols: *3 weeks is different from 6 weeks; **3 weeks is different from 9 weeks; ***3 weeks is different from both 6 and 9 weeks; †6 weeks is different from 9 weeks.

using FlowCount™ Fluorospheres and gating on TCRαβ$^+$ cells. Three-week-old rats had fewer TCRαβ$^+$CD4$^-$CD8$^-$, TCRαβ$^+$CD4$^+$CD8$^+$, TCRαβ$^+$CD4$^+$CD8$^-$ and TCRαβ$^+$CD4$^-$CD8$^+$ compared to both 6- and 9-week-old rats.

*Blood*. Figure 7d shows the absolute number of T lymphocyte subpopulations in the total blood volume using FlowCount™ Fluorospheres and gating on TCRαβ$^+$ cells. Three-week-old rats had fewer TCRαβ$^+$CD4$^-$CD8$^-$, TCRαβ$^+$CD4$^+$CD8$^+$,

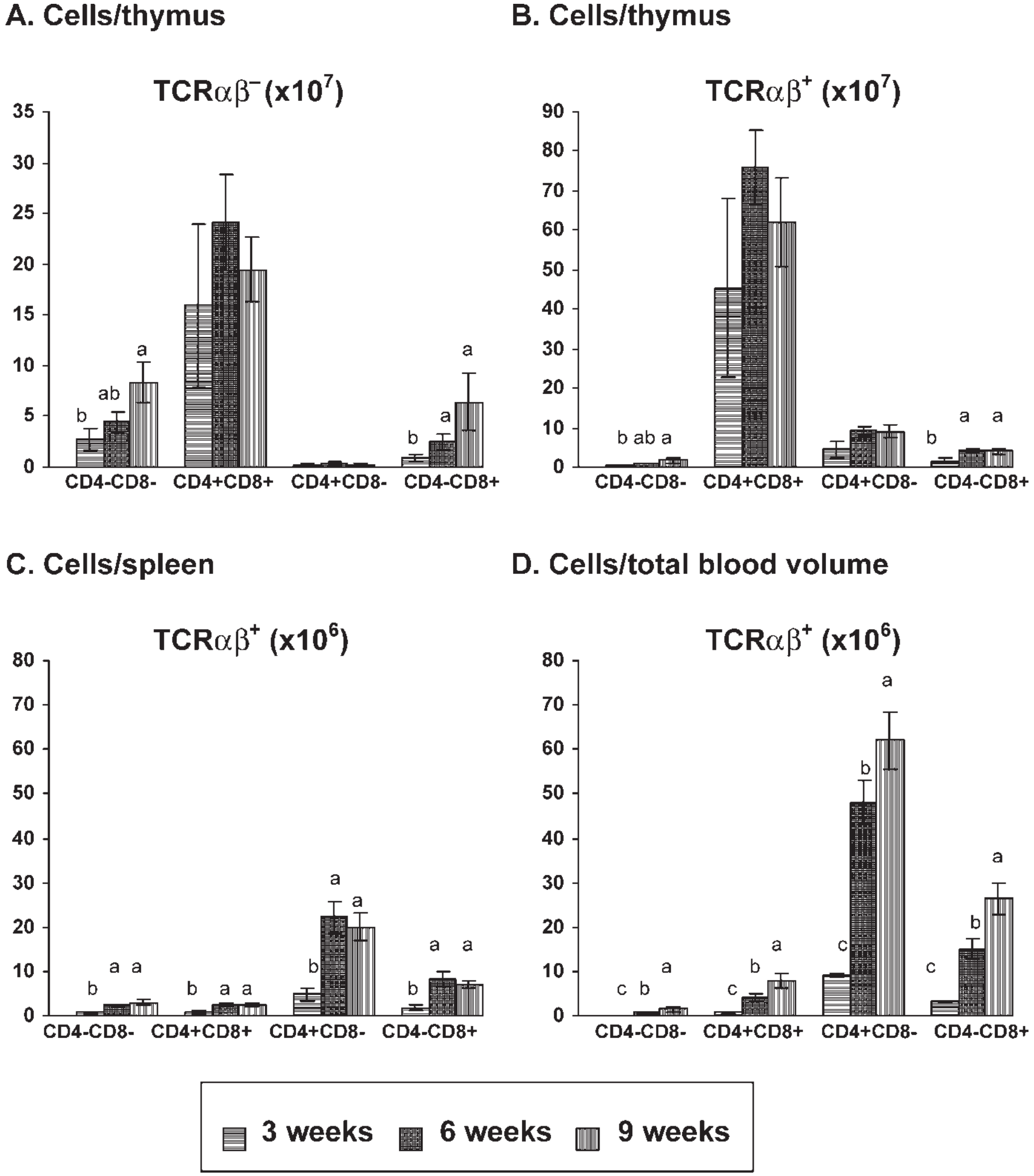

Figure 7. Absolute number of cells per thymus (a and b), spleen (c) and total blood volume (d) of young rats. Values are mean ± standarderror for $n = 9$ (3 weeks), $n = 8$ (6 weeks) and $n = 6$ rats (9 weeks). Different superscript letters indicate statistical differences ($p < 0.05$) among age groups.

$TCR\alpha\beta^+CD4^+CD8^-$ and $TCR\alpha\beta^+CD4^-CD8^+$ cells compared to 6-week-old rats, and 6-week-old rats had fewer cells of each phenotype compared to 9-week-old rats.

### Discussion

In the present study, $p56^{lck}$ protein levels decreased with age in thymic lymphocytes, but increased with age in splenic lymphocytes in 3–9-week-old Sprague-Dawley rats (Figure 4). $p56^{lck}$ is involved in the maturation of double negative (DN) to double positive (DP) thymocytes (Abraham et al. 1991, Molina et al. 1992, Levin et al. 1993, Zamoyska et al. 2003). There was a higher percentage of DN ($TCR\alpha\beta^-CD4^-CD8^-$) cells and a lower percentage of DP ($TCR\alpha\beta^-CD4^+CD8^+$) cells in the thymus of 9-week-old rats compared to 3-week-old rats

(Figure 6) which coincides with the decrease in thymic p56$^{lck}$ levels. Previous work with cultured cells and transgenic mice has shown that strong TCR signals amplified by p56$^{lck}$ contributes to CD4 or CD8 lineage choice (Mitnacht et al. 1998, Sohn et al. 2001). Preferential differentiation of DP to CD4 in mice and to CD8 in rats has been reported when cells from both species are stimulated *in vitro* (Mitnacht et al. 1998). In the present study, 9-week-old rats had a higher proportion of thymic single positive CD8 cells compared to 3-week-old rats. This may be a reflection of higher thymocyte p56$^{lck}$ protein levels in the younger rats and the time course for T cell maturation. Alternatively, the *in vitro* conditions may not reflect all the influences of the *in vivo* whole body environment. Future studies should determine the level of p56$^{lck}$ in each cell population to confirm these observations.

There were only minor differences among the age groups in the proportion of T cell subsets in the spleen and blood indicating that the age-related changes in the thymus were not reflected in the periphery. Others have investigated T cell maturation in the rat during the first year of life and found that in the thymus DN cells increased while DP cells decreased with age, along with no changes in T cell subsets in the blood (Capri et al. 2000). The ages of rats in the present study correspond to the period of rapid growth and dietary changes due to stages of life (post-lactation). The weanling rat is frequently used for investigation of dietary and environmental factors.

Three-week-old rats had a lower number of TCR$\alpha\beta^+$ cells per organ and per gram of spleen and $\mu$l of blood compared to 6- and 9-week-old rats (Figure 7). This suggests that 3-week-old rats are less equipped to respond to antigens and might leave them more susceptible to infection. However, when TCR$\alpha\beta^+$ cells were corrected for body weight there are no differences among age groups in the thymus or spleen indicating that the T cell numbers are maintained proportional to body weight in the growing rat (Figure 5).

### Acknowledgements

Author would like to thank staff of the Animal Holding Facility and Amy Noto for their assistance with animal care. Funding was provided by the Natural Sciences and Engineering Research Council of Canada to CGT and the Canadian Institutes of Health Research for maintenance support of the Faculty of Medicine's Flow Cytometry Laboratory.

### References

Totey SM, Kukarni AB. 2000. Gene targeting in immunology. Indian J Exp Biol 38:733–745.

Ravirajan CT, Isenberg DA. 2002. Transgenic models of tolerance and autoimmunity: With special reference to systemic lupus erythematosus. Lupus 11:843–849.

Wong FS, Wen L. 2003. The study of HLA class II and autoimmune diabetes. Curr Mol Med 3:1–15.

Woodward B. 1998. Protein, calories, and immune defenses. Nutr Rev 56:S84–S92.

Shou J, Ross S, Koeppen H, de Sauvage FJ, Gao WQ. 2001. Dynamics of notch expression during murine prostate development and tumorigenesis. Cancer Res 61:7291–7297.

Kannagi M, Ohashi T, Hanabuchi S, et al. 2000. Immunological aspects of rat models of HTLV type 1-infected T lymphoproliferative disease. AIDS Res Hum Retrov 16:1737–1740.

Colombo LL, Lopez MC, Chen GJ, Watson RR. 1999. Effect of short-term cocaine administration on the immune system of young and old C57BL/6 female mice. Immunopharmacol Immunotoxicol 21:755–769.

Zamoyska R, Basson A, Filby A, Legname G, Lovatt M, Seddon B. 2003. The influence of the src-family kinases, Lck and Fyn, on T cell differentiation, survival and activation. Immunol Rev 191:107–118.

Abraham KM, Levin SD, Marth JD, Forbush KA, Perlmutter RM. 1991. Delayed thymocyte development induced by augmented expression of p56$^{lck}$. J Exp Med 173:1421–1432.

Molina TJ, Kishihara K, Siderovski DP, et al. 1992. Profound block in thymocyte development in mice lacking p56$^{lck}$. Nature 357:161–164.

Levin SD, Anderson SJ, Forbush KA, Perlmutter RM. 1993. A dominant-negative transgene defines a role for p56$^{lck}$ in thymopoiesis. EMBO J 12:1671–1680.

Sohn SJ, Forbush KA, Pan XC, Perlmutter RM. 2001. Activated p56$^{lck}$ directs maturation of both CD4 and CD8 single-positive thymocytes. J Immunol 166:2209–2217.

Glaichenhaus N, Shastri N, Littman DR, Turner JM. 1991. Requirement for association of p56$^{lck}$ with CD4 in antigen-specific signal transduction in T cells. Cell 64:511–520.

Lepage LM, Giesbrecht JC, Taylor CG. 1999. Expression of T lymphocyte p56$^{lck}$, a zinc-finger signal transduction protein, is elevated by dietary zinc deficiency and diet restriction in mice. J Nutr 129:620–627.

Hosea HJ, Rector ES, Taylor CG. 2003. Zinc-deficient rats have fewer recent thymic emigrant (CD90+) T lymphocytes in spleen and blood. J Nutr 133:4239–4242.

Sharon J. 1998. Basic Immunology. Baltimore: Williams and Williams.

Reeves PG, Nielsen FH, Fahey GC. 1993. AIN-93 purified diets for laboratory rodents: Final report of the American Institute of Nutrition *ad hoc* writing committee on the reformulation of the AIN-76A rodent diet. J Nutr 123:1939–1951.

Mitnacht R, Bischof A, Torres-Nagel N, Hünig T. 1998. Opposite CD4/CD8 lineage decisions of CD4+8+ mouse and rat thymocytes to equivalent triggering signals: correlation with thymic expression of a truncated CD8$\alpha$ chain in mice but not rats. J Immunol 160:700–707.

Capri M, Qualino D, Verzella G, et al. 2000. A cytofluorimetric study of T lymphocyte subsets in rat lymphoid tissues (thymus, lymph nodes) and peripheral blood: A continuous remodeling during the first year of life. Exp Gerontol 35:613–625.

Olfer ED, Cross BM, McWilliam AA. 1993. Guide to the Care and Use of Experimental Animals. Vol. 1. Ottawa: Canadian Council on Animal Care.

